# Insulin-like growth factor system components expressed at the conceptus-maternal interface during the establishment of equine pregnancy

**DOI:** 10.3389/fvets.2022.912721

**Published:** 2022-09-13

**Authors:** Charlotte Gibson, M. de Ruijter-Villani, Tom A. E. Stout

**Affiliations:** Department of Clinical Sciences, Faculty of Veterinary Medicine, Utrecht University, Utrecht, Netherlands

**Keywords:** equine, pregnancy, insulin-like growth factor, endometrium, conceptus, asynchronous embryo transfer

## Abstract

In many species, the insulin-like growth factors (IGF1 and IGF2), their receptors and IGF binding proteins play important roles in preparing the endometrium for implantation, and regulating conceptus growth and development. To determine whether the IGF system may contribute to conceptus-maternal interaction during equine pre-implantation development, we evaluated mRNA expression for IGF system components in conceptuses, and endometrium recovered from pregnant and cycling mares, on days 7, 14, 21 and 28 after ovulation. We also investigated expression of IGF1, IGF2 and their receptors 6 and 11 days after transfer of day 8 embryos to synchronous (day 8) or asynchronous (day 3) recipient mares. Expression of *IGF1* and *IGF2, IGF1R, IGF2R, INSR* and *IGFBPs 1, 2, 4* and *5* was evident in endometrium and conceptus membranes during days 7–28. Endometrial *IGF2, INSR, IGFBP1* and *IGFBP2* expression increased between days 7 and 28 of pregnancy. In conceptus membranes, expression of all IGF system components increased with developmental stage. Immunohistochemistry revealed strong expression of IGF1, IGF2 and IGF1R in both endometrium and conceptus membranes, whereas INSR was highly expressed in endometrium but barely detectable in the conceptus. Finally, a negatively asynchronous uterine environment retarded *IGF1, IGF2* and *INSR* expression in the conceptus, whereas in the endometrium only *INSR* expression was altered by asynchrony. The presence of IGFs, their receptors and IGFBPs in the endometrium and conceptus during early equine pregnancy, and down-regulation in the conceptus following asynchronous embryo transfer, suggest a role in conceptus-maternal communication during the preparation for implantation.

## Introduction

The insulin-like growth factor (IGF) system is thought to play important roles in endometrial preparation for successful implantation, and in embryo and placental growth and development. The IGF system comprises two principal growth factors, IGF1 and IGF2, which regulate growth, cell differentiation, migration and proliferation, and inhibit apoptosis *via* paracrine and autocrine pathways (1). The action of the IGFs is mediated by the IGF receptor type 1 (IGF1R) and the insulin receptor (INSR), whereas the IGF receptor type 2 (IGF2R) regulates IGF2 availability by internalization and degradation (1, 2). In murine pregnancy, deletion of any of IGF1, IGF2 or IGF1R leads to fetal growth retardation and reduced placental size (3–5), and IGF1 has been proposed to regulate placental nutrient exchange (5, 6). The IGFs also have high affinity for the IGF binding proteins 1–6 (IGFBP1–6) which regulate IGF1 and IGF2 availability by binding to them in a way that extends their circulatory half-life, either by sequestration or by regulating their release to the receptors in target tissues (1, 7). In the endometrium of women (4, 5, 8), rodents (3) and ruminants (9–13), all members of the IGF system are expressed, and the system is thought to stimulate endometrial cell differentiation and thereby aid preparation for implantation (8, 14).

Expression of some IGF family members has been studied during early equine pregnancy. *IGF2* mRNA expression has been reported in equine placental membranes and the embryo/fetus between days 20 and 150 of gestation, having first appeared in the vascularized extra-embryonic mesoderm of the day 20 conceptus (15); the authors speculated that IGF2 might help promote invasiveness of the trophoblast cells in the chorionic girdle, and be involved with more general development of the placenta. Furthermore, *IGF2* is an imprinted gene known to be paternally expressed in equine trophoblast, and therefore expected to promote conceptus growth (16). Reports of *IGF2* expression in the endometrium are contradictory, in that an early study failed to detect *IGF2* during the first half of pregnancy (15) whereas a subsequent article reported expression of *IGF2* mRNA from day 11 of gestation (17). IGF1 has been reported to be secreted by both equine endometrium and the conceptus and is detectable in uterine luminal fluids (ULF), the endometrium and the blastocoel cavity (18). From as early as day 8 of pregnancy, *IGF1* mRNA is expressed in the endometrium and the conceptus (17, 19), while the protein has been detected in the trophectoderm and endoderm of day 12 conceptuses (19) as well as in the placental microcotyledons during the second half of gestation (20). Although *IGF1* expression by endometrium and/or conceptus was not affected by treatment with the progestogen, altrenogest, *in vivo* (17) or by estradiol 17β (E2) *in vitro* (18), the high concentrations of IGF1 detected during early equine pregnancy suggest that it may have a function during this period. Arai et al. (20) suggested that IGF1 may be involved in placentation because IGF1 expression in the microcotyledons increased at the time of chorioallantoic-endometrial interdigitation. Recently, transcriptomic studies have indicated that mRNA for other members of the IGF system are expressed in early equine conceptus membranes and pregnant endometrium. In the endometrium, *IGFBPs 1-3* were up-regulated on day 12 compared to day 8 of pregnancy (21), and *IGFBP1* expression was higher on day 13.5 of pregnancy than the equivalent day of the estrous cycle (22). In the conceptus, *IGF1R* and *IGF2R* were up-regulated between days 8 and 12 of pregnancy *and IGFBP 2, 4* and *6* expression increased between days 8 and 14 (23). IGF2R, a maternally expressed imprinted gene, is expressed in equine trophoblast (16) and detected in blastocoel fluid and culture medium conditioned by day 9 and 10 equine conceptuses (24). Soluble IGF2R is an important carrier of IGF2 and mannose 6-P proteins during pregnancy, and serves as an antagonist to the cellular receptor by opposing the growth stimulating effects of IGF2 (25).

Equine pregnancy is characterized by a long pre-implantation period, with fixation of the conceptus occurring at around day 16–17 after ovulation, but true interdigitation between the chorion and the endometrial epithelium only starting at around day 40 (26). Another interesting characteristic of horse pregnancy is the ability of the equine embryo to tolerate considerable negative uterine asynchrony (i.e., being transferred to the uterus of a recipient that ovulated after the donor mare). Although increasing the degree of negative asynchrony up to 5 days does not seem to interfere with the establishment and maintenance of pregnancy, it causes a delay in subsequent conceptus development that is visible at macroscopic, transcriptomic, cellular and tissue levels (27–29). This makes asynchronous ET an interesting model for examining factors proposed to be involved in conceptus-maternal dialogue during the establishment of pregnancy. While P4 secreted by the corpus luteum is not the only factor responsible for stimulating the endometrium to prepare for its roles in implantation and support of conceptus development (30), it is one of the most important. Following negatively asynchronous embryo transfer, the duration of endometrial exposure to P4 is shorter than following synchronous transfer, for any given embryonic stage. While this abbreviated period of endometrial P4 priming undoubtedly contributes to subsequent retarded conceptus development, it is not yet clear how the conceptus senses the stage of the endometrium and consequently adapts its development, or which factors or signals regulate this interaction (29). However, we recently found that asynchronous transfer results in consistently higher expression of *KNG1* and *IGFBP3* in the endometrium of synchronous compared to asynchronous pregnancies on days 14 and 19 of embryo development, suggesting a role for both in matching conceptus development to uterine stage (31).

Given the importance of the IGF system for regulating conceptus development and preparing the endometrium for implantation in a range of mammalian species, we hypothesized that the IGFs, their receptors and binding proteins would be involved in pre-implantation equine conceptus-maternal interaction and play a role in the growth retardation evident after transferring embryos to an asynchronous uterus. To investigate these hypotheses, we first examined the expression of members of the IGF system in the embryo/conceptus and endometrium from pregnant and cycling mares. Next, we examined changes in expression of IGF system components after transfer of day 8 embryos into the uterus of synchronous (day 8) or negatively asynchronous (day 3) recipient mares.

## Materials and methods

### Animals

Animal procedures were approved by Utrecht University's Animal Experimentation Committee (permits 2007.III.02.036 and 2012.III.02.020). Material was recovered from two groups of 18 (study 1) and 22 (study 2) warmblood mares, aged 4–15 years and maintained on pasture. When the mares were in late estrus, follicle development was monitored daily transrectally using an ultrasound scanner equipped with a 7.5-MHz transducer (MyLab30Vet; Esaote, Maastricht, The Netherlands). When pregnancy was required, estrous mares with a dominant follicle ≥35 mm were inseminated with ≥500 x 10^6^ sperm cells from a fertile stallion, and ovulation was confirmed within 48 h of insemination in all mares. Non-pregnant mares required as embryo recipients or for endometrial biopsy, were monitored identically, but were not inseminated. For ET, the desired degree of (a)synchrony between donor and recipient mares was ensured by using exogenous hormones to induce luteolysis (PGF2α analog: 37.5 mg D-cloprostenol; Genestranvet: Eurovet Animal Health B.V., Bladel, The Netherlands) and ovulation (hCG: 1500 IU Chorulon; Intervet, Boxmeer, The Netherlands). Day 7 or 8 pregnancy was confirmed by blastocyst recovery during uterine lavage, whereas later conceptus vesicle presence was detected, and development monitored, by transrectal ultrasonography.

### Study design and tissue collection

Study 1 involved investigation of mRNA expression for *IGF1, IGF2, IGF1R, IGF2R, INSR, IGFBPs 1, 2, 4* and *5*, and of protein expression for IGF1, IGF2, IGF1R and INSR in conceptus membranes and endometrium collected during early pregnancy (days 7, 14, 21 and 28) and across the estrous cycle (days 7, 14 and 21 after ovulation). Despite numerous attempts, we were unable to validate primers to investigate *IGFBP3* expression. On days 14, 21 and 28 of gestation, conceptuses (*n* = 4 per group) were collected using an endoscopically-guided net, after puncture of the membranes and aspiration of the conceptus fluid with a sharpened PTFE catheter, as described previously (32). Endometrial biopsies were recovered from the base of a uterine horn using uterine biopsy forceps on days 7, 14 and 21 after ovulation in cycling mares, and days 7 and 14 in pregnant mares; on days 21 and 28 of pregnancy, the biopsy was recovered from the site of conceptus apposition (visible as a hyperaemic area adjacent to the punctured membranes) under endoscopic guidance (*n* = 4 per group).

Study 2 investigated the effect of asynchronous ET on endometrial and conceptus expression of *IGF1, IGF2, IGF1R, IGF2R* and *INSR*. Twenty day 8 blastocysts were collected from donor mares by uterine lavage and transferred to synchronous (day 8; ovulated on the same day as the donor) or asynchronous (day 3; ovulated 5 days after the donor) recipient mares as described previously (29, 31). Resulting conceptuses (*n* = 5 per group) were recovered 6 or 11 days after ET (day 14 or 19 of conceptus development), and an endometrial biopsy (*n* = 5 per group) was recovered from the base of a uterine horn (day 6 after ET) or at the site of conceptus apposition (day 11 after ET).

After recovery and washing in 0.9 % NaCl, the embryonic disc (day 14) or embryo proper (day 19 and 21) were dissected from the membranes (yolk sac) using a stereomicroscope (Olympus SZ-ST; Olympus, Tokyo, Japan); for day 28 conceptuses, the embryo proper, yolk sac (YS) and allantochorion (AC) were separated. All tissues were then divided; one piece was snap-frozen in liquid nitrogen and stored at −80°C prior to RNA extraction, while the other piece was fixed overnight in 4% paraformaldehyde before embedding in paraffin in preparation for immunohistochemistry.

### RNA extraction and cDNA synthesis

Total RNA was extracted using the AllPrep DNA/RNA/Protein Mini kit following the manufacturers' instructions (Qiagen, Venlo, The Netherlands). Samples (30 mg) were homogenized in 600 μl lysis buffer and RNA was eluted with 40 μl RNAse-free water. cDNA synthesis was performed on 1 μg of DNASE I treated RNA (37°C and 10 min at 65°C; 1 IU/μg; RNase-free DNase kit; Qiagen) using Superscript III (Invitrogen, Landsmeer, The Netherlands) in a final volume of 20 μl.

### Quantitative PCR

Quantitative PCR was performed as described previously (29). Briefly, primer pairs were designed using PerlPrimer 1.1. (http://perlprimer.sourceforge.net/) the product specificity of the primer pairs (Eurogentec: Seraing, Belgium; Supp. Table 1) was assessed by DNA sequencing (ABI PRISM 310 Genetic analyzer; Applied Biosystem, Foster City, USA). For each gene, a 10-fold serial dilution of the target gene PCR product, of known quantity, was amplified simultaneously with the samples to produce a standard curve and thereby allow subsequent quantification. PCR was carried out in 15 μl reaction mixture containing 1 μl sample cDNA and iQ SYBR® Green Supermix, on an IQ5 real-time PCR system (Bio-Rad Laboratories; Veenendaal, The Netherlands). Cycle conditions were: 3 min denaturation at 95°C, followed by 40 cycles of amplification (15 s at 95°C, 30 s at the primer-specific annealing temperature and 30 s at 72°C). This was followed by 1 min at 95°C, 1 min at 55°C, and finished with a melting curve. Product specificity was evaluated by assessing the melting curve using iQ5 software, and starting quantities were quantified using the standard curve. Relative gene expression was calculated as the ratio of target gene mean expression to the geometric mean for the housekeeping genes selected for the specific tissue and experiment from 4 candidates (GAPDH, PGK1, HPRT1 and SRP14), after stability evaluation using GeNorm (33). Potential housekeeping genes had to have a stability value (M) below 0.5. The 4 genes were ranked in terms of the lowest M value and the 2 most stable genes were selected for normalization; additional genes were included in a stepwise fashion as long as overall stability was not affected. For endometrium in study 1 and conceptus membranes in study 2, the combination of GADPH, PGK1 and SRP14 was found to be optimal for normalization, whereas for conceptus membranes in study 1 and endometrium in study 2 GAPDH, PGK1, HRPT1 and SRP14 were selected.

**Table 1:** Primer pairs for equine IGF system components; gene symbol, primer sequence, annealing temperature, product size and accession number of genes used for qRT-PCR analysis.

**Gene**	**Primer sequence (5' → 3') F: forward R: reverse**	**Ta (°C)**	**Product size (bp)**	**Accession number**
***IGF1***	F: ACGCTCTTCAGTTCGTGTGT	60	137	NM_001082498.2
	R: CAGCCTCCTCAGATCACAGC			
***IGF2***	F: GGACCGCGGCTTTTACTTCA	62	121	NM_001114539.1
	R: GGTGGCACAGTAGGTCTCCA			
***IGF1R***	F: ATGTACTTCGCTTTCAATCCC	60	290	XM_023651179.1
	R: ATCCTGCCCATCATACTCTG			
***INSR***	F: CGAGTTGGATTATTGCCTCAAAG	62	101	XM_001496584.3
	R: CGTACTCACTCTGATTGTGCTTCTG			
***IGF2R***	F: GTAACGCTAAGCTTTCCTATT ACG	60	188	XM_005608119.1
	R: GGTATACCACCGGAAGTTGTA GG			
***IGFBP1***	F: TTATCTGCCAAACTGCAACAAGA	62	85	XM_014738877.1
	R: CCAGCAGAGCCCCACTTCT			
***IGFBP2***	F: CTACTCCTTGCACATTCCCAACT	57	74	XM_023642295.1
	R: CGTTCAGAGACATCTTGCACTGT			
***IGFBP4***	F: CGGAGATCGAGGCTATCCAG	58	139	XM_003362485.2
	R: CTCCGGTCTCGAATTTTGGC			
***IGFBP5***	F: CGACCGCAAGGGATTCTACA	60	141	XM_001490309.4
	R: AAGGTGTGGCACTGGAAGTC			
***GAPDH***	F: AGGCCATCACCATCTTCCAG	53	112	NM_001081838.1
	R: ACCGGAGTCCATCACGATGC			
***HPRT1***	F: GAGATGTGATGAAGGAGATGG	58	232	XM_001490189.2
	R: CTTTCCAGTTAAAGTTGAGAGG			
***PGK1***	F: CTGTGGGTGTATTTGAATGG	54	151	XM_001502668.3
	R: GACTTTATCCTCCGTGTTCC			
***SRP14***	F: CTGAAGAAGTATGACGGTCG	55	101	XM_001503583.2
	R: CCATCAGTAGCTCTCAACAG			

### Immunohistochemistry

Immunohistochemistry was performed as described previously (34). Briefly, 5 μm sections cut from paraffin-embedded tissue were mounted on SuperFrost® Plus slides (VWR International, Leuven, Belgium). After the deparaffinization and rehydration steps, a 1% H_2_O_2_ solution in methanol was used to block endogenous peroxidase activity. Antigen retrieval was performed by microwaving (750 W) the sections for 15 min, in pre-heated Tris/EDTA-buffer for IGF1, IGF1R and IGF2 (0.01 M Tris; 0.001 M EDTA; 0.05% Tween-20; pH 9.0), or with citrate buffer for INSR (10 mM citric acid; pH 6.0). Slides were cooled to room temperature (RT) over 30 min and rinsed in PBS-Tween (PBST; 0.05% Tween-20 in PBS: 3 x 5 min). To block non-specific binding, the sections were incubated for 15 min with goat serum, for IGF1, IGF1R and INSR (1:10 in PBS), or rabbit serum for IGF2 (1:10 in PBS). Next, sections were incubated at 4°C overnight with the specific antibody (Table 2) and, after rinsing with PBST, sections were incubated with the second antibody diluted 1:250 for 30 min at RT (Table 2). Sections were then incubated with Avidin-Biotin-Complex (ABC)-peroxidase for 30 min (Vectastain® ABC Kit, PK-4000; Vector Laboratories), followed by a 10 min incubation in freshly prepared 3,3'-diaminobenzidine tetrahydrochloride (45 ml 0.05 M Tris/HCl, pH 7.6; 5 ml DAB and 5 μl H_2_O_2_), and counterstained with hematoxylin for 30 s. Finally, the sections were dehydrated and mounted under a coverslip with Eukitt™ Mounting Medium (Electron Microscopy Systems, Hatfield, USA). Imaging was performed using an Olympus BX60 microscope (Olympus Nederland, Leiderdorp, the Netherlands) coupled to a digital camera (Leica DFC425C: Leica Microsystems, Wetzlar, Germany) and Leica LAS-AF software. For negative controls, the primary antibody was substituted with purified immunoglobulin (IgG; Table 2).

**Table 2:** List of antibodies and IgGs used for immunohistochemistry of equine endometrium and conceptus membranes.

**Protein**	**Primary antibodies**	**References (dilution)**	**Secondary antibodies**	**References**	**IgG for control (dilution)**
**IGF1**	Rabbit polyclonal anti-IGF1	H70 sc-9013 Santa Cruz (1: 66)	Biotinylated goat anti-rabbit	BA-1000, Vector Laboratories Inc.	Rabbit IgG (1: 5000)
**IGF2**	Goat polyclonal anti-IGF2	F20 sc-7435 Santa Cruz (1: 50)	Biotinylated rabbit anti-goat	BA-5000, Vector Laboratories Inc.	Goat IgG (1: 100)
**IGF1R**	Rabbit polyclonal anti-IGF1R β-subunit	C20 sc-713 Santa Cruz (1: 333)	Biotinylated goat anti-rabbit	BA-1000, Vector Laboratories Inc.	Rabbit IgG (1: 200)
**INSR**	Mouse monoclonal anti-INSR β-subunit	ab69508 Abcam (1: 333)	Biotinylated goat anti-mouse	BA-9200, Vector Laboratories Inc.	Mouse IgG (1: 200)

IgG, immunoglobulin.

All primary antibodies were obtained from Huissen, The Netherlands.

### Statistical analysis

Data were analyzed using SPSS 20 for Windows (SPSS Inc., Chicago, IL, USA). QRT-PCR data was log transformed to yield normally distributed data sets. Endometrial gene expression was then analyzed by two-way ANOVA, followed by *post-hoc* Tukey testing. If a significant interaction between pregnancy status and stage of pregnancy/cycle was apparent, data were sub-divided according to status or stage and reanalyzed by one-way ANOVA. Conceptus membrane gene expression was analyzed by one-way ANOVA with *post-hoc* Tukey testing. Data from the ET study was analyzed by two-way ANOVA to investigate effects of pregnancy stage and synchrony; significant effects were verified by independent *T*-tests. Statistical significance was assumed when *P* < 0.05.

## Results

### Gene expression for IGF system components in the endometrium of cycling and pregnant mares

**Figure 1:**
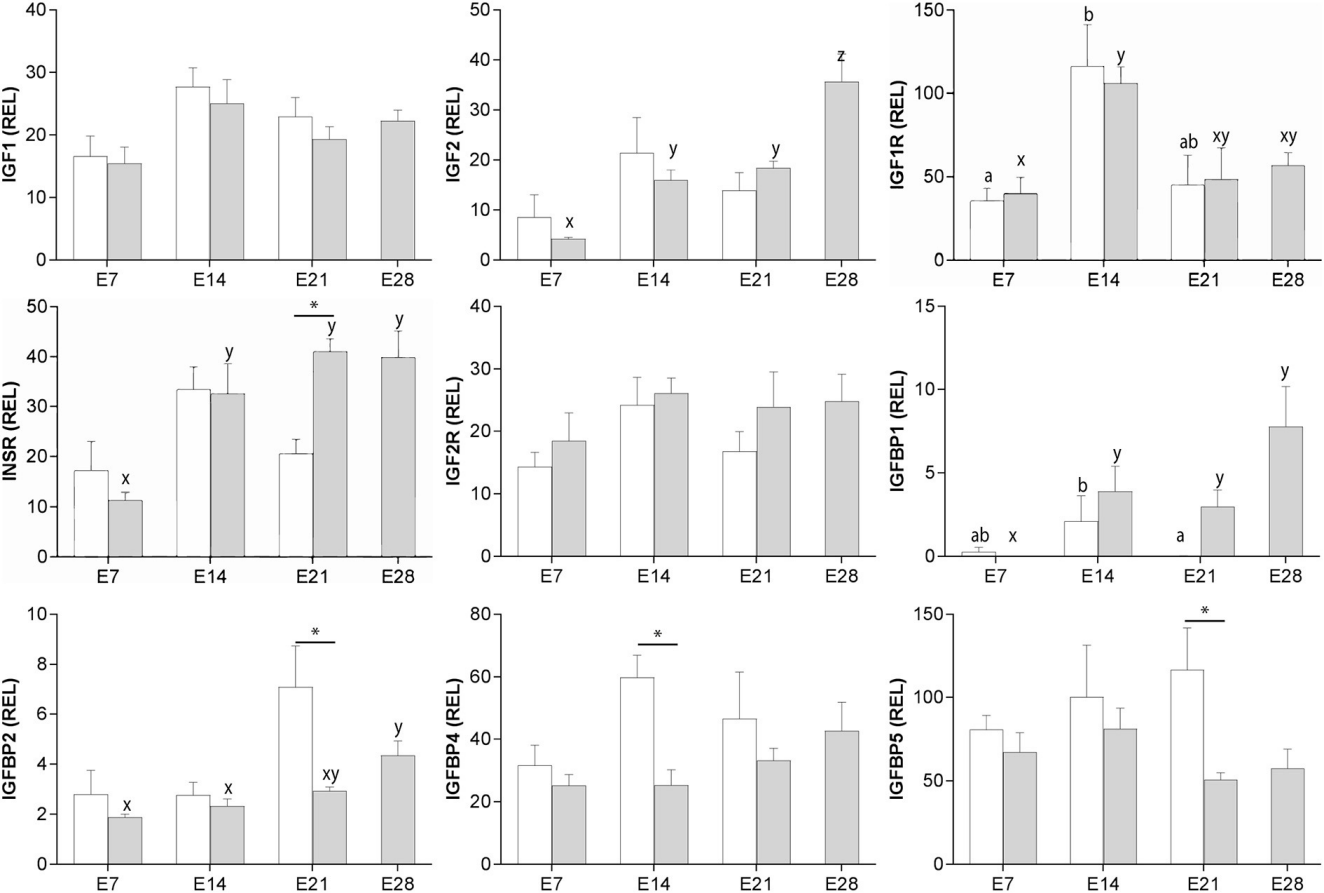
Relative gene expression (mean ± s.e.m) for *IGF1, IGF2, IGF1R, INSR, IGF2R, IGFBP1, IGFBP2, IGFBP4* and *IGFBP5* in equine endometrium from cyclic (white bars) or pregnant mares (gray bars) on days 7, 14, 21 and 28 (pregnancy only) after ovulation. Values are calculated as the ratio of the target gene mean value to the geometric mean for the reference genes *GAPDH, PGK1* and *SRP14*. Significant differences between condition (cyclic vs. pregnant) within the same day are indicated by an asterisk (*P* < 0.05), while differences between days are denoted by different superscripts (cycle: a, b; pregnancy; x, y, z; *P* < 0.05).

*IGF1* and *IGF2R* expression did not vary significantly across the estrous cycle and early pregnancy (Figure 1). Expression of *IGF2* did not change markedly during the cycle, but in pregnant animals increased in two steps between days 7 and 28 of pregnancy (P < 0.001 and P < 0.05, respectively; Figure 1). *IGF1R* expression increased only between days 7 and 14 in both cycling and pregnant mares (*P* < 0.05; Figure 1). *INSR* expression increased after day 7 of pregnancy (*P* < 0.001), and was twice as high on day 21 of pregnancy as day 21 of the cycle (i.e., estrus; *P* < 0.01; Figure 1). *IGFBP1* expression decreased between days 14 and 21 of the cycle (*P* < 0.05), and increased after day 7 of pregnancy (*P* < 0.01). *IGFBP1* expression was higher in endometrium from pregnant mares than that of mares in estrus (day 21 of the cycle) (*P* < 0.001; Figure 1). Conversely, *IGFBP2, IGFBP4* and *IGFBP5* were more highly expressed in endometrium from day 21 cycling (estrus) than pregnant mares (*P* < 0.05; Figure 1). Nevertheless, IGFBP2 expression increased between days 14 and 28 of pregnancy (*P* < 0.01).

### Gene expression for IGF system components in early equine conceptus membranes

**Figure 2:**
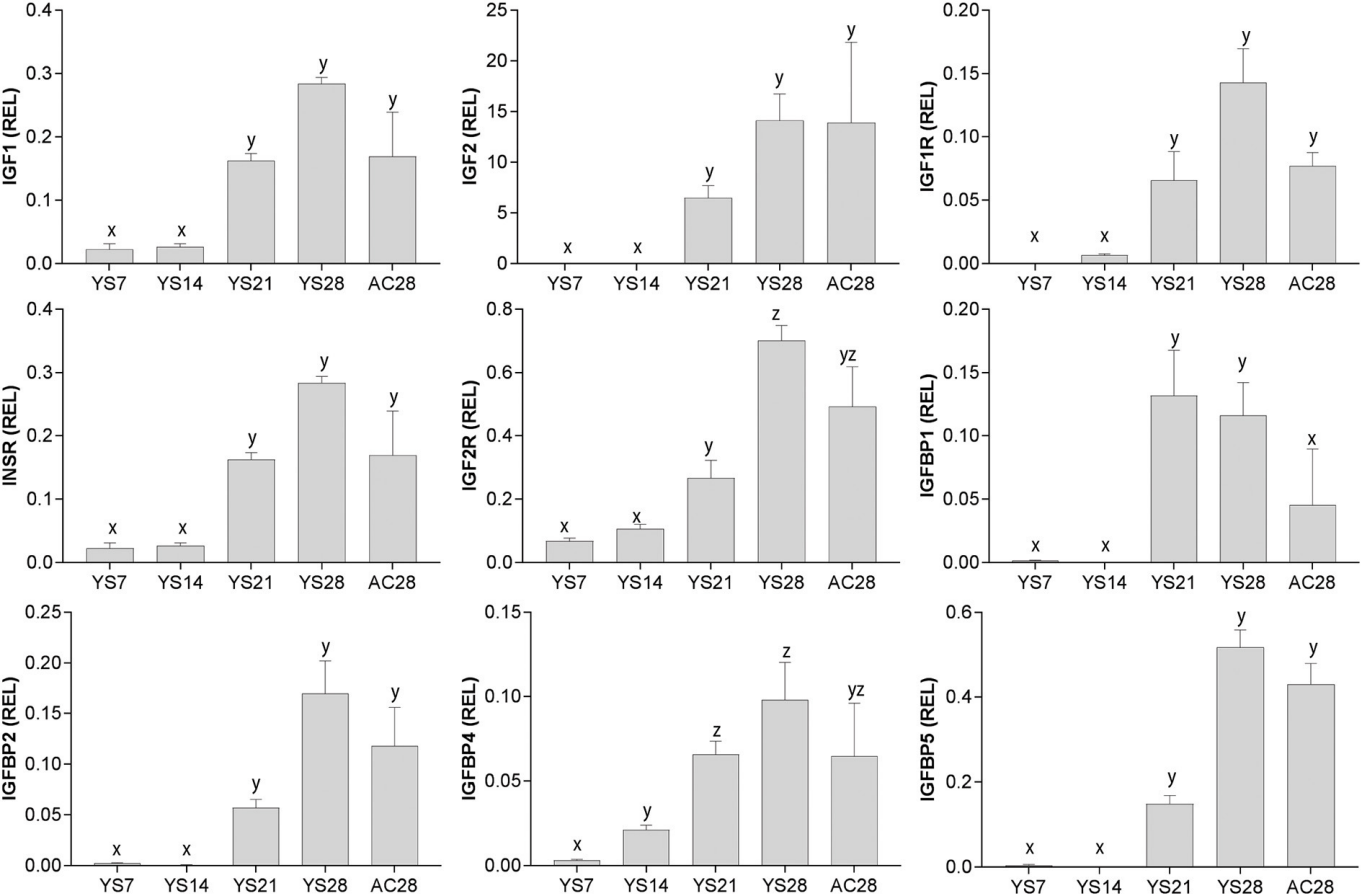
Relative gene expression (mean ± s.e.m) for *IGF1, IGF2, IGF1R, INSR, IGF2R, IGFBP1, IGFBP2, IGFBP4* and *IGFBP5* in equine conceptus membranes (yolk sac: YS; allantochorion: AC) on different days of pregnancy. Values are calculated as the ratio of the target gene mean value to the geometric mean for the reference genes *GAPDH, HPRT1, PGK1* and *SRP14*. Significant differences between pregnancy stages are denoted by different superscripts (x, y, z; *P* < 0.05).

The expression of mRNA for all of the genes studied increased with the advance of early pregnancy in yolk sac (YS), in particular after day 14 (*P* < 0.05; Figure 2), and was relatively high in allantochorion (AC) once this membrane had developed.

### Localization of IGF system proteins in equine endometrium

**Figure 3:**
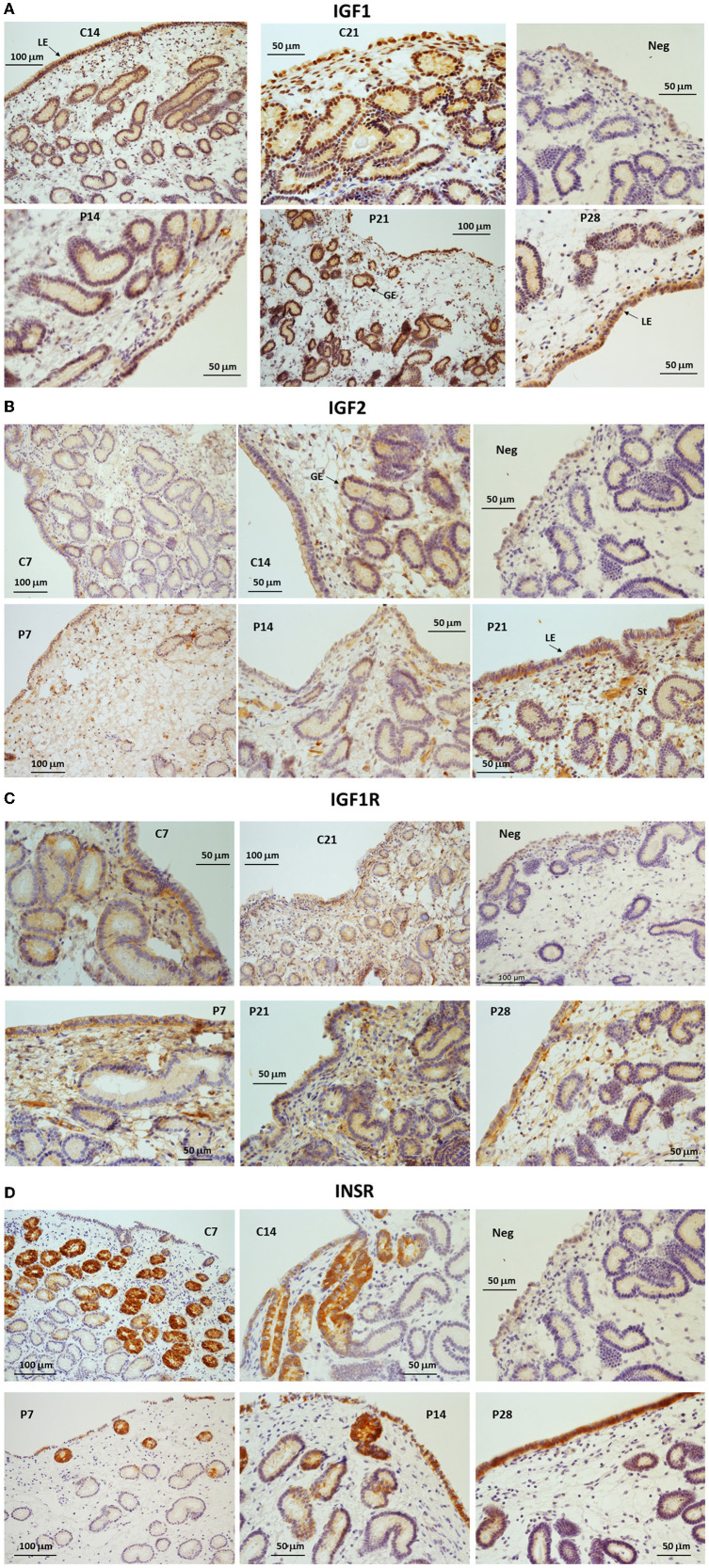
**(A–D)** Immunohistochemical localization of **(A)** IGF1, **(B)** IGF2, **(C)** IGF1R and **(D)** INSR in endometrium from cyclic (C: days 7, 14 or 21) and pregnant (P: days 7, 14, 21 or 28) horse mares. NC, negative control. LE, luminal epithelium; GE, glandular epithelium; St, stroma.

Immunostaining for IGF1 was detectable in the cytoplasm of luminal epithelial cells (LE) and glandular epithelial cells (GE; Figure 3A) in endometrial samples from all cycling and pregnant mares. During the cycle, a strong signal was detected in the basal part of the LE on day 14, whereas on day 21 (estrus) the signal was mainly detected on the apical aspect of the LE (Figure 3A). During pregnancy, IGF1 was abundant in the GE and LE on day 21 of pregnancy, whereas on day 28 IGF1 staining was mainly localized to the apical side of the LE. IGF1 protein was not detected in the stroma (Figure 3A). IGF2 immunostaining was weak on day 7 of the cycle but subsequently increased in the LE, GE and stroma. On days 7 and 14 of pregnancy, weak IGF2 staining was evident in the LE, GE and the stroma (Figure 3B). From day 21 of pregnancy, moderate IGF2 staining was detected in the LE and stroma (Figure 3B). IGF1R protein was localized to the GE, stroma and the LE with strong staining apparent in the basal part of the LE on day 7 of the cycle and pregnancy (Figure 3C). By day 21, the staining was more diffuse within the LE, GE and stroma, with a stronger intensity in endometrium from pregnant mares. On day 28, IGF1R staining was stronger in the apical and basal aspects of the LE. INSR was abundant in the GE on day 7 of diestrus but staining intensity had reduced by days 14 and 21 when some staining appeared in the LE (Figure 3D). On day 28 of pregnancy, INSR expression was not detectable in the GE, but was moderately intense in the LE (Figure 3D).

### Localization of IGF system proteins in early equine conceptus membranes

**Figure 4:**
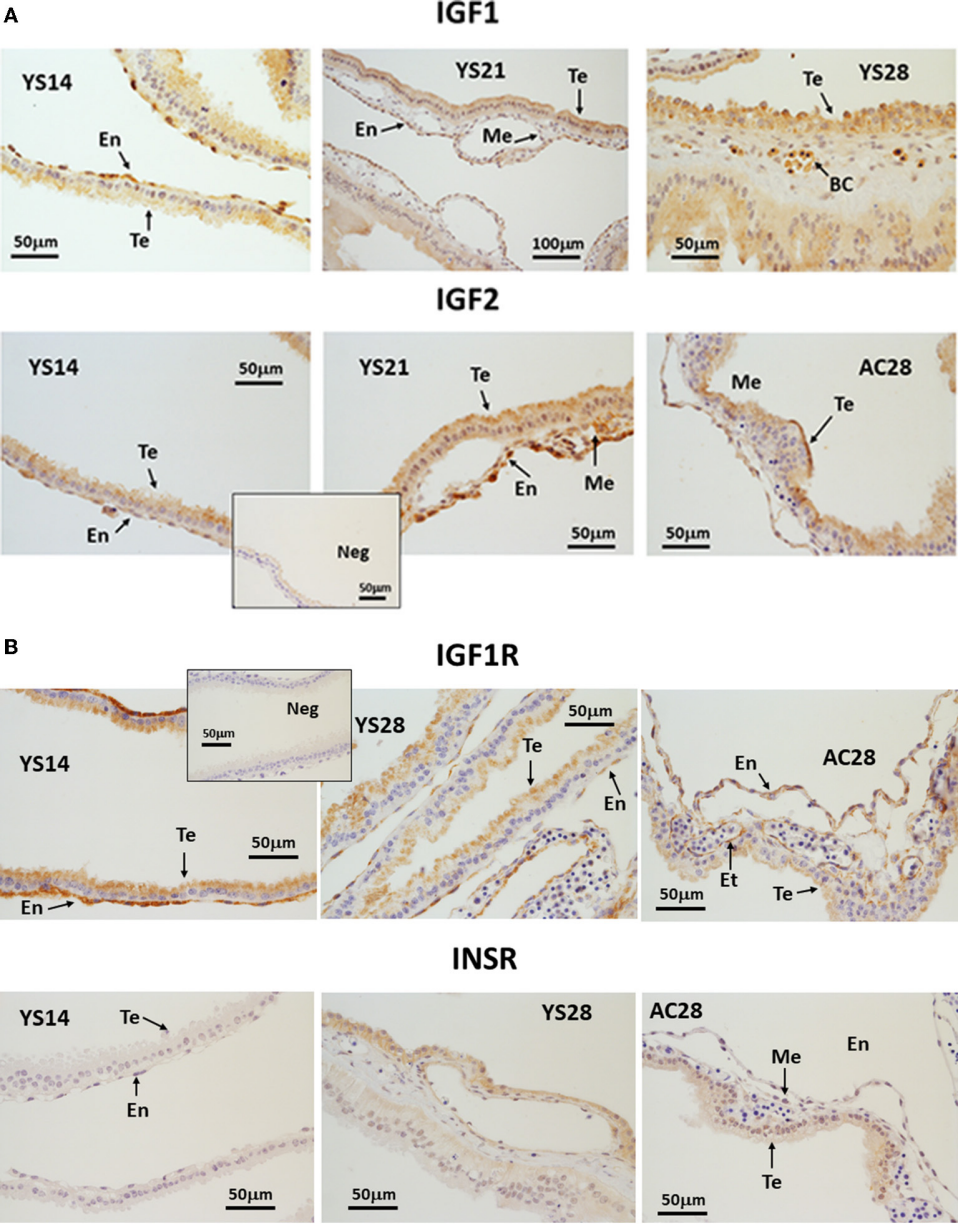
**(A, B)** Immunohistochemical localization of **(A)** IGF1 and IGF2, **(B)** IGF1R and INSR in equine conceptus membranes on days 14, 21 and 28 of pregnancy (YS, yolk sac; AC, allantochorion). Te, trophectoderm; En, endoderm; Me, mesoderm; BC, blood cells; Et, endothelium.

IGF1 was detected from day 14 of pregnancy in trophectoderm (Te) and endoderm cells (En) and staining intensity increased in the apical part of the Te on days 21 and 28 (Figure 4A). Furthermore, IGF1-stained nucleated cells were visible within the blood vessels from day 21. IGF2 immunostaining was moderate in Te and En cells at all stages. IGF2 protein was more abundant on day 21 of pregnancy, from when it could also be detected in the mesoderm (Me). By day 28, the signal was strongest in the apical part of the Te (Figure 4A). IGF2 was not detected in endothelial cells. IGF1R protein staining was intense in the En and apical part of the Te, with weak staining appearing in the endothelium from day 21. On day 28, the staining of the En remained strong in the YS but was weaker in the AC (Figure 4B). Finally, staining for INSR was absent on days 14 and 21 and only weak staining was detected in the En and Me of day 28 YS and in the Te of AC (Figure 4B).

### Impact of asynchronous ET on IGF system component mRNA expression

**Figure 5:**
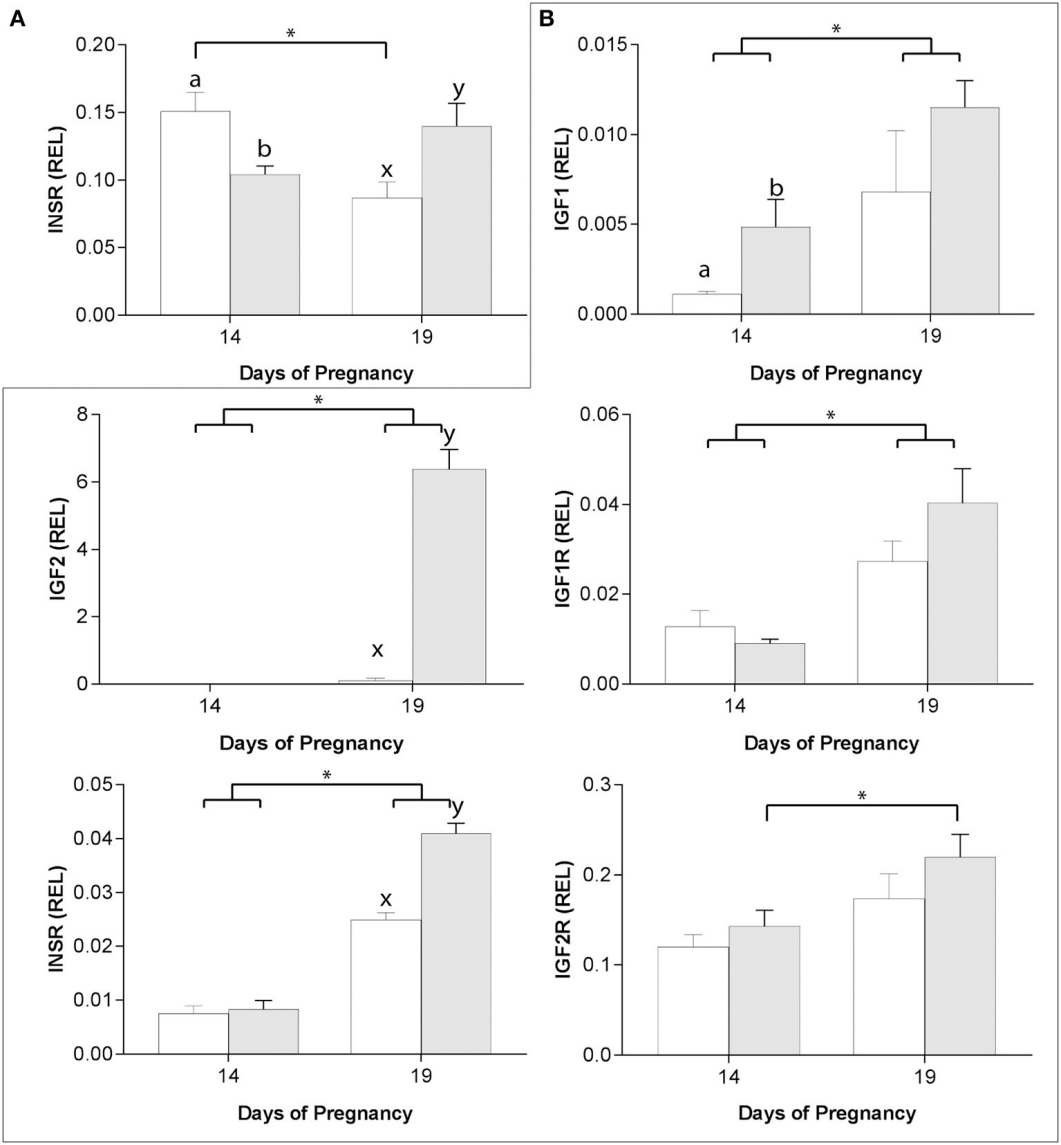
Relative gene expression (mean ± s.e.m) on days 14 and 19 of pregnancy after transfer of day 8 horse embryos to asynchronous (day 3: white bars) or synchronous (day 8: gray bars) recipient mares, for *INSR* in the endometrium **(A)** and for *IGF1, IGF2, IGF1R, INSR*, and *IGF2R* in the conceptus membranes **(B)**. Values are calculated as the ratio of the target gene mean value to the geometric mean for the reference genes (*GAPDH, PGK1* and *SRP14* for the conceptus, and *GAPDH, HPRT1, PGK1* and *SRP14* for the endometrium). Significant differences (*P* < 0.05) between group (asynchronous vs. synchronous) within the same day are depicted by different superscripts (day 14: a, b; day 19: x, y) while pregnancy stage differences within a group are indicated by an asterisk (*).

In the endometrium, only *INSR* expression was affected by asynchronous ET (Figure 5A), with expression higher 6 days after asynchronous than synchronous ET but lower 11 days after asynchronous ET (day 19 of embryo development). In addition, while *INSR* expression increased from day 14 to day 19 of pregnancy after synchronous ET, it decreased between days 14 and 19 in asynchronous pregnancies (Figure 5A).

In previous studies, we showed that the expression of the placental imprinted genes *IGF2, INSR* and *IGF2R* was affected by asynchronous ET (29). In the present study, we found that *IGF1* and *IGF1R* expression was also affected by asynchronous ET. On day 14 of conceptus development, *IGF1* was more highly expressed in YS after synchronous than asynchronous ET, with expression increasing between days 14 and 19 in both groups (*P* < 0.05; Figure 5B). *IGF1R* and *INSR* expression increased between days 14 and 19 of conceptus development in both groups (*P* < 0.05) and, on day 19, expression for *INSR* was higher in synchronous than asynchronous conceptuses (*P* < 0.001; Figure 5B). *IGF2* was not detectable in day 14 YS in either group, and was higher in synchronous pregnancies on day 19 (*P* < 0.01; Figure 3B). Finally, *IGF2R* mRNA transcript increased between days 14 and 19 only in YS from synchronous conceptuses (*P* < 0.05; Figure 5B).

## Discussion

This study demonstrated that most IGF system components are expressed in the endometrium of cycling and pregnant mares and in embryos and the conceptus membranes between days 7 and 28 of pregnancy, and are differentially regulated during the estrous cycle and pregnancy. *IGF1* and *IGF2, IGF1R, IGF2R* and *INSR* and *IGFBP 1, 2, 4* and *5* mRNA was detected in the endometrium and in conceptus membranes. In addition, IGF1, IGF2 and IGF1R protein was expressed strongly in the endometrium and the conceptus, whereas INSR was expressed primarily in the endometrium. Transferring an embryo to an asynchronous recipient retarded the upregulation of gene expression for *IGF1, IGF2* and *INSR* in the yolk sac membrane, but altered the gene expression of only *INSR* in the endometrium.

Previous publications reported measurable quantities of IGF1 in uterine fluids, the endometrium and in blastocoel fluid (18) during early equine pregnancy, and found that both mRNA and protein were detectable in the endometrium and conceptus membranes (17, 19). We confirmed that IGF1 is produced by the endometrium and the conceptus between days 7 and 28 of pregnancy. However, equine endometrial expression of *IGF1* does not seem to be regulated by P4 or conceptus factors, since mRNA levels were stable across both the cycle (including estrus) and early pregnancy and were not affected by uterine-conceptus asynchrony. Similarly, *IGF1* expression in equine endometrium was not affected by treating mares with the synthetic progestogen, altrenogest (17), nor was it influenced *in vitro* by exposing endometrial explants to E2 (18). This contrasts to reports in mice and pigs, in which both E2 and P4 have been shown to stimulate uterine *IGF1* expression (35, 36). Although we did not see any increase in *IGF1* mRNA expression, we did find that IGF1 protein expression was relocated to the apical aspect of the luminal epithelium and the glandular epithelium during pregnancy. This suggests that IGF1 produced by the LE and the endometrial glands could be secreted more effectively into the uterine lumen, where it is better placed to influence conceptus development. Alternatively, IGF1 produced in the GE could act in a paracrine fashion on IGF1R expressed in surrounding endometrial cells to stimulate uterine function and thereby indirectly support conceptus development (13). In conceptus membranes, IGF1 mRNA and protein increased during pregnancy with expression evident in the TE, En, and nucleated blood cells. In rabbit blastocysts, IGF1 has a mainly mitogenic role inducing cell proliferation and growth, and promoting development of the embryonic disc (37, 38).

In contrast to *IGF1, IGF2* expression in the endometrium did appear to be regulated by prolonged exposure to P4 and/or the conceptus, given that it increased during pregnancy but not diestrus. The intensity of IGF2 protein staining also increased during pregnancy, and it was highly expressed in the apical aspect of the LE on day 28, which suggests that it could be secreted into the uterine lumen to support conceptus growth. Previously, it was reported that IGF2 was expressed only from day 20 of pregnancy in the vascularized mesoderm, and that its expression increased in the allantochorion after implantation (15). However, we found IGF2 to be highly expressed in the trophectoderm, endoderm and mesoderm from day 14 of pregnancy. IGF2 could play a role in equine conceptus growth and placentation and, in particular, it has been proposed to play a role in cell migration and invasion because it is more highly expressed in the invasive cells of the chorionic girdle than in non-invasive trophoblast prior to implantation (15); in this respect, IGF2 is known to stimulate trophectoderm cell migration in sheep (39).

In endometrium, *IGF1R* expression increased on day 14 of the cycle and pregnancy, whereas protein expression was more intense on day 21 in both conditions, primarily in the stroma and the LE. It is possible that P4 is responsible for stimulating *IGF1R* expression, as described for human endometrial stromal cells (40), with protein expression maintained in day 21 cycling mares despite the return to estrus; alternatively, E2 produced by the follicle and/or the early conceptus may play a role in stimulating increased IGF1R expression following a period of P4 dominance. The increased abundance of IGF1R during early pregnancy would increase its availability for binding IGF1 and IGF2, and thereby presumably help promote mitogenic activity in the endometrium as part of the preparation for implantation (40). Previously, Klein et al. (23) found that *IGF1R* was up-regulated in the early equine conceptus and, here, we show that IGF1R is expressed in the apical part of the trophectoderm, the endoderm, and in endothelial cells. IGF1 has a higher affinity for IGF1R than IGF2, but both are apparently able to activate IGF1R in mouse embryos since deletion of the receptor results in a more severe growth retardation than deletion of either IGF1 or IGF2 (3, 41–43). It is likely that both endometrial IGF1 and IGF2 exert any actions on the equine conceptus *via* IGF1R (44). Furthermore, IGF1 was expressed by nucleated cells within the blood vessels while IGF1R was present in endothelial cells, therefore IGF1 could be involved in stimulating blood vessel formation and development in equine conceptus membranes *via* the IGF1R, as reported in other species (45, 46).

In this study, we found an increase in *INSR* expression during pregnancy with stage-specific changes in localization of the protein; initially, INSR was primarily detected in glandular epithelium but, between days 21 and 28, expression switched to the luminal epithelium. In addition, *INSR* was the only member of the IGF family to be differentially expressed in endometrium between synchronous and asynchronous pregnancies, with lower expression in asynchronous than synchronous pregnancies on day 19 of conceptus development. This either means that *INSR* requires a longer duration of P4 exposure for increased expression, or that factors expressed by a more advanced conceptus are involved in stimulating or maintaining expression. In the rat, INSR is expressed in the LE and stromal cells and is upregulated primarily in the LE at the site of implantation (47). In women, *INSR* is expressed in epithelial and stromal cells, and a treatment designed to mimic decidualization in human endometrial cells induced INSR up-regulation (40, 48). Since IGF2 has a high affinity for both the INSR homodimer and the IGF1R/INSR heterodimer, and the activation of these receptors by IGF2 triggers cellular proliferation and differentiation (16), these results suggest that endometrial INSR plays an important role in the preparation for implantation. While *INSR* mRNA was also detected in conceptus membranes, protein levels were very low and detectable only on day 28 of pregnancy, suggesting that any INSR does not play a direct role in conceptus development early in pregnancy.

Expression of the *IGF2R* and *IGF2* genes in equine trophoblast has been shown to be imprinted, with *IGF2R* preferentially expressed from the maternal allele and *IGF2* from the paternal allele (16). Both are involved in regulating conceptus growth and development, with deletion of IGF2R leading to placental and fetal overgrowth, whereas deletion of IGF2 leads to placental and fetal growth restriction, in mice (5, 41, 42, 48). In equine conceptuses, *IGF2R* mRNA was previously reported to be up-regulated between days 8 and 12 of pregnancy (23), and IGF2R protein was secreted by day 9 and 10 conceptuses (24). Our results support these previous studies, with conceptus membrane *IGF2R* expression also increasing as pregnancy progressed. IGF2R presumably plays a role in regulating IGF2 abundance to optimize fetal growth. On the other hand, it has been reported that IGF2 is able to stimulate cell migration mediated via IGF2R in bovine endothelial cells (49) and human extravillous trophoblast cells (43, 50).

IGFBP1 is one of the most studied IGFBPs during early pregnancy. In the horse, P4 and the presence of a conceptus appear to stimulate endometrial *IGFBP1* expression, and E2 to supress, since *IGFBP1* increased during pregnancy but decreased between days 14 and 21 of the cycle (late diestrus and estrus, respectively). Endometrial *IGFBP1* has also been reported to be up-regulated between days 8 and 12 of pregnancy (21) and to be more highly expressed on day 13.5 of pregnancy than the cycle (22), with the respective authors suggesting that IGFBP1 could regulate IGF1 trafficking between the endometrium and the uterine luminal fluid. Similarly, in ruminants IGFBP1 is stimulated by interferon-tau, P4 and prostaglandins (9, 51, 52), and has been shown to stimulate ovine trophoblast cell migration and attachment (11, 51). In women, IGFBP1 is a major secretary product of decidual cells, a marker for decidualization and involved with modulation of trophoblast invasion (7, 53). In equine pregnancy, IGFBP1 has been proposed to play a role in conceptus-endometrium communication and in particular in modulating implantation by regulating trophoblast invasion (51, 54). *IGFBP2* mRNA levels increased during estrus and later equine pregnancy (day 28), suggesting that its expression could be stimulated by E2 and/or the presence of the conceptus; IGFBP2 expression also increases during pregnancy in the pig and responds to E2 and P4 in human endometrial cells (54). While IGFBP4 and IGFBP5 were expressed in equine endometrium, they increased during estrus rather than pregnancy. In equine conceptus membranes, *IGFBP1, 2, 4* and *5* mRNA expression increased during early pregnancy; however, protein expression and localization is not yet known. Previously, *IGFBP4* was found to be up-regulated between days 8 and 12 of pregnancy (23), and the literature supports the idea that elevated IGFBP1 in the fetus is linked to growth restriction (4, 41, 55), suggesting that IGFBP1 could help regulate IGF1 availability in the conceptus or directly affect placental development. The functions of IGFBPs 2, 4 and 5 have been less studied, but as they are expressed in the endometrium and the conceptus, they presumably bind IGF1 and IGF2 and regulate their availability and/or have direct effects in preparing the endometrium or trophectoderm for implantation. The present study did not include *IGFBP3* simply because, despite a number of attempts, we were unable to design successful PCR primers. However, in a related study in which next generation sequencing was used to examine the effects of asynchronous embryo transfer on the endometrial transcriptome, we found that *IGFBP3* was consistently upregulated in the endometrium of synchronous compared to asynchronous pregnancy (31). Other transcriptomic studies have similarly indicated that *IGFBP3* expression is upregulated in the endometrium of pregnant compared to non-pregnant mares (21, 22), while IGFBP3 protein has been detected in the uterine luminal fluids and associated to the blastocyst capsule (56). In studies designed to examine a direct effect of IGFBP3 on trophectoderm cells, we were unable to demonstrate a clear effect on cell proliferation or attachment to plastic *in vitro* (31), and are therefore currently left to speculate that it plays the proposed role of matching conceptus and endometrial stage indirectly, e.g., by regulating availability of the IGFs, as suggested for other IGFBPs.

In the conceptus, *IGF1, IGF2, IGF1R* and *INSR* increased between days 14 and 19 after both synchronous and asynchronous ET, whereas *IGF2R* only increased after synchronous ET. In addition, *IGF2* and *INSR* were less highly expressed in day 19 yolk sac after asynchronous than synchronous ET. The reduced *IGF2* and *INSR* expression in conceptus membranes after asynchronous ET is presumably a result of the associated delay in conceptus development. Interestingly, *INSR* expression was lower in the endometrium of asynchronous mares, and this decrease in *INSR* is presumably either a result of inadequate stimulation from the retarded conceptus or may contribute to the delay of conceptus development. In this respect, reduced availability of INSR could affect the ability of insulin and the IGFs to activate pathways *via* their receptors and IGFBP1 (57, 58).

In conclusion, members of the IGF system are highly expressed in both the endometrium and conceptus membranes during early equine pregnancy and are likely to play important roles in conceptus growth and development before implantation, and in preparing the endometrium to support implantation. IGF1 and IGF2 are expressed by the endometrium and the conceptus and presumably exert their effects *via* IGF1R and INSR in the endometrium and IGF1R in the conceptus membranes; these effects could include stimulating cell proliferation and differentiation. The expression of IGF1 and IGF1R in the conceptus membranes and the presence of IGF1 protein in blood cells suggests a role in vascularization and the development of endothelial cells. In the endometrium, although IGF1R is abundant, increased INSR expression in the luminal epithelium on day 28 of pregnancy implies a specific role in preparing for implantation.

## Data availability statement

The raw data supporting the conclusions of this article will be made available by the authors, without undue reservation.

## Ethics statement

The animal study was reviewed and approved by Utrecht University's Animal Experimentation Committee (permits 2007.III.02.036 and 2012.III.02.020).

## Author contributions

CG performed the practical work, analysis and wrote the first draft of the manuscript. MDR-V contributed to project planning, performance, and supervision. TS involved in funding acquisition, supervision, and edited the manuscript. All authors have read and agreed to the final version of the manuscript.

## Funding

This research was supported by the European Commission under the Marie Curie ITN project EpiHealthNet; FP7-PEOPLE-2012-ITN n° 317146.

## Conflict of interest

The authors declare that the research was conducted in the absence of any commercial or financial relationships that could be construed as a potential conflict of interest.

## Publisher's note

All claims expressed in this article are solely those of the authors and do not necessarily represent those of their affiliated organizations, or those of the publisher, the editors and the reviewers. Any product that may be evaluated in this article, or claim that may be made by its manufacturer, is not guaranteed or endorsed by the publisher.
